# High Prevalence of Skin Disorders among HTLV-1 Infected Individuals Independent of Clinical Status

**DOI:** 10.1371/journal.pntd.0002546

**Published:** 2013-11-07

**Authors:** Renata Okajima, Augusto C. P. Oliveira, Jerusa Smid, Jorge Casseb, Jose Antonio Sanches

**Affiliations:** 1 HTLV-Outpatient Clinic, Institute of Infectious Diseases “Emilio Ribas,” São Paulo, Brazil; 2 Department of Dermatology, University of São Paulo Medical School, São Paulo, Brazil; 3 Institute of Tropical Medicine of São Paulo, Laboratory of Medical Investigation 56 (LIM56), University of São Paulo Medical School, São Paulo, Brazil; University of Washington, United States of America

## Abstract

**Background:**

Human T-cell lymphotropic virus type 1 (HTLV-1) infection can increase the risk of developing skin disorders. This study evaluated the correlation between HTLV-1 proviral load and CD4^+^ and CD8^+^ T cells count among HTLV-1 infected individuals, with or without skin disorders (SD) associated with HTLV-1 infection [SD-HTLV-1: xerosis/ichthyosis, seborrheic dermatitis or infective dermatitis associated to HTLV-1 (IDH)].

**Methods:**

A total of 193 HTLV-1-infected subjects underwent an interview, dermatological examination, initial HTLV-1 proviral load assay, CD4^+^ and CD8^+^ T cells count, and lymphproliferation assay (LPA).

**Results:**

A total of 147 patients had an abnormal skin condition; 116 (79%) of them also had SD-HTLV-1 and 21% had other dermatological diagnoses. The most prevalent SD-HTLV-1 was xerosis/acquired ichthyosis (48%), followed by seborrheic dermatitis (28%). Patients with SD-HTLV-1 were older (51 vs. 47 years), had a higher prevalence of myelopathy/tropical spastic paraparesis (HAM/TSP) (75%), and had an increased first HTLV-1 proviral load and basal LPA compared with patients without SD-HTLV-1. When excluding HAM/TSP patients, the first HTLV-1 proviral load of SD-HTLV-1 individuals remains higher than no SD-HTLV-1 patients.

**Conclusions:**

There was a high prevalence of skin disorders (76%) among HTLV-1-infected individuals, regardless of clinical status, and 60% of these diseases are considered skin disease associated with HTLV-1 infection.

## Author Summary

HTLV-1 infection may increase the risk of developing skin disorders. A total of 193 HTLV-1 infected subjects were studied, including asymptomatic carriers and HAM/TSP patients. Of the subjects, 76% had an abnormal skin condition, with a high prevalence both among HTLV-1 asymptomatic carriers and HAM/TSP patients. The most prevalent SD-HTLV-1 was xerosis/acquired ichthyosis (48%), followed by seborrheic dermatitis (28%). Patients with SD-HTLV-1 were older (51 vs. 47 years), had a higher prevalence of myelopathy/tropical spastic paraparesis (HAM/TSP) (75%) and an increased first HTLV-1 proviral load compared with patients without SD-HTLV-1. When excluding HAM/TSP patients, the first HTLV-1 proviral load of SD-HTLV-1 individuals remains higher than no SD-HTLV-1 patients. Thus, skin diseases are highly prevalent among HTLV-1-infected individuals.

## Introduction

Adult T-cell leukemia/lymphoma (ATLL), HTLV-1-associated myelopathy/tropical spastic paraparesis (HAM/TSP) and infective dermatitis associated with HTLV-1 (IDH) are the main diseases caused by human T-cell lymphotropic virus type 1 (HTLV-1) infection [1]–[3]. However, several other clinical conditions have been associated with this viral infection, such as uveitis, thyroiditis, arthritis and polymyositis [4]–[6].

There are an estimated 5 to 10 million HTLV-1 infected individuals worldwide and Brazil is considered a highly endemic area for HTLV-1 infection, with the largest absolute number of HTLV-1 infected individuals, with more than one million people living with this virus [7]–[9]. Despite this high prevalence, only a few studies on the dermatological aspects of HTLV-1 infection have been described in this country [10].

There is a lack of surrogate markers to assess the infected patients who have a higher risk for HTLV-1 associated skin disorders. Moreover, there are few immunological studies among HTLV-1-infected persons who are simultaneously suffering from HAM/TSP and skin diseases. The aim of this study is to evaluate the prevalence of skin disorders in HTLV-1-infected individuals and to correlate this prevalence with the initial HTLV-1 proviral load, and initial CD4^+^ and CD8^+^T cell count.

## Materials and Methods

In the last 18 years, a cohort of HTLV-infected subjects has been followed in the HTLV-outpatient clinic at the Institute of Infectious Diseases “Emilio Ribas” (IIER), with the support of nurses, nutritionists and physical therapists. From a total 450 HTLV-1-infected individuals, including asymptomatic carriers and HAM/TSP patients, 193 of them were consecutively evaluated for skin disorders from January 2008 to July 2010 by the same dermatologist, blinded for the clinical status to minimize information bias. Demographical and clinical dates were collected, and dermatological examinations were carried out. The HIV co-infected individuals were excluded, but HCV co-infected subjects were included.

Ethics Statement: Written informed consent was obtained from all participants, and the IIER ethical board approved the protocol.

The patients underwent laboratory studies, including HTLV-1 serological diagnosis, initial CD4^+^ and CD8^+^T cell counts, and an initial HTLV-1 proviral load. In accordance with previous studies, the following skin disorders associated with HTLV-1 infection (SD-HTLV-1) were considered: xerosis/acquired ichthyosis, seborrheic dermatitis and infective dermatitis associated with HTLV-1 (IDH) [1], [10]–[12]. HAM/TSP was diagnosed according to previously established criteria [13]. Skin culture and punch skin biopsies were performed when clinical examination was not sufficient for a dermatological diagnosis. Antibodies to HTLV-1/2 were detected by a diagnostic enzyme-linked immunosorbent assay (ELISA) and confirmed by Western blot analysis and polymerase chain reaction (PCR), which are capable of discriminating between HTLV-1 and HTLV-2 [14]. To determine the counts of CD4^+^ and CD8^+^ T-cell subsets, fresh whole blood specimens were collected in EDTA tubes and subjected to flow cytometry (Coulter EPICS® XL-MCLÔ Flow Cytometer - Beckman Coulter, Fullerton, CA), using human monoclonal antibodies anti-CD3, anti-CD4, and anti-CD8, labeled with fluorochrome.

The results of the first HTLV-1 proviral load were available in the database. Quantitative proviral DNA levels were detected by a real-time automated PCR method, using TaqMan probes for the *pol* gene. The albumin gene served as the internal genomic control, and MT2 cells were used as a positive control. The results are reported as copies/10000 PBMCs, and the detection limit was 10 copies [15].

Data were analyzed using SPSS 17.0 software. The association between independent variables and the outcome was analyzed either by Student's t-test or ANOVA (normal distribution variables), or by Mann-Whitney test (non-normal distribution variables), while the association between categorical variables and the outcome was assessed by the X^2^ test. HTLV-1 proviral load was log-transformed to obtain a normal distribution. Correlations between HTLV-1 proviral load and SD-HTLV were performed using Spearman's rank correlation. Data are expressed as mean ± standard deviation (normal distribution variables) or median and interquartile range (non-normal distribution variables). Statistical significance was set at a p value<0.05.

## Results

One hundred ninety-three HTLV-1-infected subjects (43% of all HTLV-1-infected patients at the HTLV outpatient clinic from the Emilio Ribas Institute cohort) underwent a dermatological exam. Mean age of patients was 49.4±12.3 years. Female gender had a higher prevalence of HTLV-1 infection (72%). Regarding the presence of neurological involvement, 38% of the patients had a diagnosis of HAM/TSP. The dermatological examination revealed a high prevalence of skin disorders among the HTLV-1-infected patients (76%). Sixty-five individuals (34%) had one dermatological condition, and 42% (n = 81) of the patients had two or more dermatological conditions. Among the 147 patients that had an abnormal skin condition, 79% (n = 116) had a skin disorder associated with HTLV-1 infection (SD-HTLV-1) (xerosis/ichthyosis or seborrheic dermatitis) and 21% (n = 31) had other dermatological diagnoses. The most prevalent skin disorder associated with an HTLV-1 diagnosis was xerosis/ichthyosis (48%), followed by seborrheic dermatitis (28%).

Table 1 shows the prevalence of skin disorders in the HTLV-1 patients based on a diagnosis of HAM/TSP or asymptomatic carriers. SD-HTLV-1 were more prevalent on HAM/TSP patients (xerosis/acquired ichthyosis (p = 0.007; seborrheic dermatitis (p = <0.0001); IDH (p = 0.022). Patients with SD-HTLV-1, including asymptomatic carriers and HAM/TSP, are older, have a higher prevalence of HAM/TSP, and have a higher first HTLV-1 proviral load (performed for 109 patients, p = 0.009), compared with patients without SD-HTLV-1 (Table 2). Note that 75% of the SD-HTLV-1 group was made up of HAM/TSP individuals.

**Table 1:** Dermatological findings among human T-cell lymphotropic virus type-1 (HTLV-1) infected individuals (HAM/TSP and asymptomatic carriers).

Dermatological findings	HAM/TSP N (%)	HTLV-1 Asymptomatic carriers N (%)	p value
	n = 73	n = 120	
Xerosis/Acquired ichthyosis	44 (60.2)	51 (42.5)	0.006
Seborrheic dermatitis	35 (47.9)	18 (15.0)	<0.0001
Infestations	30 (41.1)	29 (24.1)	0.007
Contact dermatitis	1 (1.4)	3 (2.5)	0.619
Melasma	3 (4.1)	3 (2.5)	0.497
Infective dermatitis associated to HTLV-1	3	0	0.022
Other diagnoses	11 (15.0)	17 (14.1)	0.771
Normal findings on dermatological exam	9 (12.3)	37 (30.8)	0.005

HAM/TSP: HTLV-1-associated myelopathy/Tropical spastic paraparesis.

Infestations: Dermatophytosis (N: 55), Scabies (N;1), Pityriasis versicolor (N;3).

Other diseases: Herpes simplex (N: 4), Pytiriasis alba (N; 3), Cheilitis (N;3), Callosity (N: 2), Pressure ulcer (N:2), Verruca vulgaris (N:2); Vitiligo (N: 1), Photosensitivity (N: 1), Hypertrichosis (n: 1), Neurofibromatosis (N;1), Insect bite reaction (N;1), Acanthosis nigricans (N:1), Lichen planus (N:1), Nummular dermatitis (N:1), Basal cell carcinoma (N:1),, Epidermal cyst (N:1), Rhinophyma (N:1), Miliaria (N:1), Rosacea (N:1), Exogenous ochronosis (N:1), Venous ulcer (N:1), Folliculitis (N:1), Lymphoma (N:1), Hyperkeratosis plantare (N:1), Keratosis pilaris (N:1), Erythrodermia (N:1), Folliculitis decalvans (N:1), Pyogenic granuloma (N:1), Livedo reticularis (N:1), Madarosis (N:1), Erythema nodosum/Panniculitis (N:1), Molluscum contagiosum (N:1).

**Table 2:** Characteristics of patients according to the presence of dermatological diseases associated to HTLV-1 infection.

Variable	Skin disorder associated to HTLV-1 (N = 116)	No skin disorder associated to HTLV-1 (N = 77)	p value
**Age (years, mean, SD)**	51±12	47±12	0.014
**Female (%)**	76	66	0.190
**HCV coinfected (%)**	13.9	14.3	1.000
**Log_10_ HTLV-1 proviral load (copies/10^4^ PBMCs, mean, SD)**	2.30±0.87*	1.75±1.00**	0.009
**T CD4 cells count (cells/mm^3^, median, interquartile range)**	1033 (766–1298)	982 (817–1285)	0.91
**T CD8 cells count (cells/mm^3^, median, interquartile range)**	557 (386–756)	548 (441–726)	0.97
**HAM/TSP (%)**	75	25	0.002

HCV: Hepatitis C virus; SD: Standard deviation; Skin disorders associated to HTLV-1: xerosis/acquired ichthyosis and seborrheic dermatitis.

* N tested = 67 subjects,

** N. tested: 42 subjects.

Table 3 depicts the presence of SD-HTLV-1 in subjects that are asymptomatic for neurological symptoms (absence of HAM/TSP). Mean age of SD-HTLV-1 patients was 51 years, as compared with 44 years for HTLV-1 patients without SD (p = 0.002), regardless of gender, CD4^+^ and CD8^+^ T cell counts (p = 0.489; p = 0.824). The initial HTLV-1 proviral load was significantly higher for the group with SD-HTLV-1 as compared with that for the group without SD-HTLV-1 and asymptomatic for neurological symptoms (p = 0.021).

**Table 3:** Presence of skin disorders, epidemiological and laboratorial characteristics of the HTLV-1-infected subjects.

Variable	Skin disorder associated to HTLV-1(N = 61)	No skin disorder associated to HTLV-1 (N = 59)	p value
**Age (years, mean, SD)**	51±13	44±12	0.002
**Female (%)**	55	45	0.167
**HCV coinfected (%)**	10.0	11.9	0.777
**Log_10_ HTLV-1 proviral load (copies/10^4^ PBMCs, mean, SD)**	2.05±0.86*	1.41±0.88**	0.021
**T CD4 cells count (cells/mm^3^, median, interquartile range)**	1035 (771.5–1318.5)	934 (814–1246)	0.38
**T CD8 cells count (cells/mm^3^, median, interquartile range)**	541 (378.25–747.75)	544 (432–703)	0.82

HCV: Hepatitis C virus; SD: Standard deviation; Skin disorders associated to HTLV-1: xerosis/acquired ichthyosis and seborrheic dermatitis.

* N: 31,

** N: 31.

## Discussion

Notably, 76% of the HTLV-1-infected asymptomatic carriers and 88% of the HAM/TSP patients showed some skin disorder in our study. These findings are similar to those described in two previous studies involving asymptomatic carriers and HAM/TSP subjects [10], [12]. Thus, it is important to stress that HTLV-1 infection may have an etiological link to skin disease. In fact, skin disorders are highly associated with HTLV-1 infection, regardless of neurological symptoms, and they may represent a clinical warning sign for the diagnosis or progression of this infection [10]–[12].

Excluding HAM/TSP cases, subjects with a diagnosis of SD-HTLV-1 are older than groups with other types of skin disorders or individuals with a normal dermatological exam. However, no significant association was observed with gender. These findings suggest that older HTLV-1-infected individuals, who probably have a longer duration of their viral infection have had more time to develop SD-HTLV-1.

Although IDH is the only skin disease in which HTLV-1 infection is a criterion for diagnosis, other skin disorders could also be associated with HTLV-1 infection, including xerosis/acquired ichthyosis and seborrheic dermatitis, as previously demonstrated [10]–[12], [16]. In fact, in our study, these illnesses were the most prevalent skin disorders associated with adult HTLV-1-infected individuals, regardless of the clinical status. However, studies with a longer follow-up should be performed to assess the hypothesis that these skin manifestations are related to HTLV-1 infection.

The exact pathogenic mechanism of IDH still needs to be made clear, but the current view is that a diagnosis of HTLV-1 infection is necessary, leading in susceptible individuals to immune deregulation, with subsequent immunosuppression and superinfection with *Staphylococcus aureus* and beta-haemolytic streptococci, what additionally leads to chronic antigenic stimulation and persistent inflammation of the skin. Genetic, host and environmental factors have been shown to be associated [17].

Xerosis and acquired ichthyosis have been described as the main dermatological manifestations associated with HAM/TSP patients [11], [12], [18]. Xerosis is characterized by dryness of the skin, and acquired ichthyosis is clinically characterized by cutaneous xerosis and the formation of polygonal thin flat scales of varying sizes, mainly on the extremities [19].

Acquired ichthyosis is a consequence of hypohydrosis that may be secondary to the involvement of the autonomic nervous system, affecting directly the HTLV-1-infected skin cells [12], [20]. On the basis of histopathological and immunohistochemical analyses of skin fragments of acquired ichthyosis from HAM/TSP individuals, it was concluded that keratinocytes are activated, probably as a result of cytokines that are liberated from HTLV-1-infected lymphocytes. This activation leads to an interference in keratinocyte differentiation and migration, resulting in a defect in the processes of normal desquamation, accumulation and retention of corneocytes [18]. We noticed that more than 50% of the SD-HTLV-1 cases involved xerosis/acquired ichthyosis and decided to include them in the same group because the acquired ichthyosis showed a similar clinical dermatological pattern, comparable with higher degree xerosis, and so they were clinically difficult to differentiate [12].

HTLV-1 proviral load is a laboratorial risk marker for the development of HAM/TSP and other diseases related to HTLV-1 infection [21], [22]. The initial HTLV-1 proviral load was higher in the group with SD-HTLV-1 (p = 0.009) as well as in the SD-HTLV-1 HAM/TSP-free group (p = 0.021), both of which were statistically significant. Although the proviral load may differ greatly among individuals, it is relatively stable during the course of the HTLV-1-related disease [23]. In HAM/TSP patients this finding suggests that proviral load reaches a stable level determined by the relationship between viral expression and the immune response against the virus [23].

As previously shown, HTLV-1 was identified by PCR on skin cells in addition to lymphocytes in HTLV-1-infected persons, regardless of their clinical status ^10^. Because of these findings, several authors believe that HTLV-1 can modify the function of infected cells, resulting in skin disorders that are caused directly by the presence of the virus in the infected cells [10], [16], [24]. Another possible mechanism of skin disorders among HTLV-1-infected individuals is the production of cytokines in HTLV-1-infected lymphocytes, promoting a functional disturbance on skin cells [18]. This lack of association may be explained by the presence of HTLV-1 in specific sites that occur during HAM/TSP and the proviral load in the cerebral spinal fluid (CSF) [25]. Lezin et al. reported that the proviral load quantified in CSF was able to distinguish clearly between healthy groups of HTLV-1 carriers and patients presenting HAM/TSP [25].

Finally, for the first time, a high prevalence of skin disorders (76%) independent of clinical status was disclosed among HTLV-1-infected individuals. These findings may have important implications in the clinical setting in places where this infection is endemic and dermatologists, infectious diseases specialists and general clinicians should be aware of skin presentations of the HTLV-1 infection. Moreover, the influences of demography and co-morbid conditions may be relevant, but have not been fully studied. Thus, data derived from a cohort of referred patients followed in a specialized HTLV clinic may not be a representative sample of the whole population of HTLV-1 patients and therefore unlikely to reflect the prevalence of skin conditions among all HTLV-1 patients.

## Supporting Information

Checklist S1 STROBE checklist.
(DOCX)
