# Supplementary material for: High Prevalence of Skin Disorders among HTLV-1 Infected Individuals Independent of Clinical Status
Source: PLoS Negl Trop Dis. 2013 Nov 7;7(11):e2546. doi: 10.1371/journal.pntd.0002546 (PMC3820737; doi:10.1371/journal.pntd.0002546)
Supplement: Checklist S1 — STROBE checklist. (DOCX) [file pntd.0002546.s001.docx]

|  | Item No | Recommendation |
| --- | --- | --- |
| **Title and abstract** | 1 | (*a*) High prevalence of skin disorders among HTLV-1-infected individuals, regardless of clinical status, regardless of clinical status. |
|  |  | (*b*) A total of 147 patients had an abnormal skin condition; 116 (79%) of these patients also had SD-HTLV-1 and 21% had other dermatological diagnoses. The most prevalent SD-HTLV-1 was xerosis/acquired ichthyosis (48%), followed by seborrheic dermatitis (28%). The patients with SD-HTLV-1 were older, had a higher prevalence of HAM/TSP (75%) and had an increased first HTLV-1 proviral load and basal LPA compared with patients without SD-HTLV-1. When excluding HAM/TSP patients, the first HTLV-1 proviral load of SD-HTLV-1 individuals remains higher than no SD-HTLV-1 patients. There was a high prevalence of skin disorders (76%) among HTLV-1-infected individuals, regardless of clinical status and 60% of these diseases are considered skin disease associated to HTLV-1 infection. |
| Introduction | | |
| Background/rationale | 2 | It is estimated that range from 5 to 10 million HTLV-1 infected individuals worldwide and Brazil is considered a highly endemic area for HTLV-1 infection with the largest absolute number of HTLV-1 infected individuals, with more than one million people living with this virus. Despite this high prevalence, only a few studies on the dermatological aspects of HTLV-1 infection have been described in this country. HTLV-1 infection can increase the risk for developing skin diseases, regardless of clinical status. Other skin diseases have being related to HTLV-1 infection, but only in case reports; few studies have addressed the HTLV-1-infected population. There is a lack of surrogate markers to assess the infected patients who have a higher risk for this condition. Moreover, there are few immunological studies among HTLV-1-infected persons who are simultaneously suffering from HAM/TSP and skin diseases. |
| Objectives | 3 | The aim of this study is to evaluate the prevalence of skin disorders in HTLV-1-infected individuals and to correlate this prevalence with the initial HTLV-1 proviral load, and initial CD4^+^ and CD8^+^T cell count. |
| Methods | | |
| Study design | 4 | Cross-sectional study |
| Setting | 5 | From a total 450 HTLV-1-infected individuals, including asymptomatic carriers and HAM/TSP, 193 of them were consecutively evaluated for skin disorders from January 2008 to July 2010 from Sao Paulo city, Brazil |
| Participants | 6 | (*a*) HTLV-1 without co-infections |
| Variables | 7 | HIV or HCV infections were excluded, only older than 18 years old |
| Data sources/ measurement | 8* | For each variable of interest, give sources of data and details of methods of assessment (measurement). Describe comparability of assessment methods if there is more than one group |
| Bias | 9 | The same dermatologist evaluated all subjects |
| Study size | 10 | 193 of them were consecutively evaluated for skin disorders from January 2008 to July 2010. |
| Quantitative variables | 11 | Explain how quantitative variables were handled in the analyses. If applicable, describe which groupings were chosen and why |
| Statistical methods | 12 | (*a*) The data were analyzed using SPSS 17.0 software. The independent variables were compared by Student's t-test and ANOVA (normal distribution variables) or Mann-Whitney test (non-normal distribution variables), and categorical variables were compared by the χ^2^ test. HTLV-1 proviral load was log-transformed to obtain a normal distribution. Correlations between HTLV-1 proviral load and SD-HTLV were performed using Spearman’s rank correlation. The data are expressed as mean ± standard deviation (normal distribution variables) or media and interquartile range (non-normal distribution variables). Statistical significance was indicated by p values <0.05. |
|  |  | (*b*) Describe any methods used to examine subgroups and interactions |
|  |  | (*c*) Explain how missing data were addressed |
|  |  | (*d*) If applicable, describe analytical methods taking account of sampling strategy |
|  |  | (*e*) Describe any sensitivity analyses |
| Results | | |
| Participants | 13* | (a) The same dermatologist evaluated all subjects during the two years |
|  |  |  |
|  |  |  |
| Descriptive data | 14* | (a) HTLV-1+, adults >18 years; |
|  |  | (b) Indicate number of participants with missing data for each variable of interest |
| Outcome data | 15* | Report numbers of outcome events or summary measures |
| Main results | 16 | (*a*) Give unadjusted estimates and, if applicable, confounder-adjusted estimates and their precision (eg, 95% confidence interval). Make clear which confounders were adjusted for and why they were included |
|  |  | (*b*) Report category boundaries when continuous variables were categorized |
|  |  | (*c*) If relevant, consider translating estimates of relative risk into absolute risk for a meaningful time period |
| Other analyses | 17 |  |
| Discussion | | |
| Key results | 18 | 76% of the HTLV-1-infected asymptomatic carriers and 88% of the HAM/TSP patients showed some skin disorder in our study. |
| Limitations | 19 | Although IDH is the only skin disease in which HTLV-1 infection is a criterion for diagnosis, other skin disorders could also be associated with HTLV-1 infection, including xerosis/acquired ichthyosis and seborrheic dermatitis. |
| Interpretation | 20 | These findings indicate as HTLV-1 potential cause of skin disease |
| Generalisability | 21 | These results are in accordance to other and show the necessity of dermatologist in the HTLV clinic |
| Other information | | |
| Funding | 22 | FAPESP |

*Give information separately for exposed and unexposed groups.

**Note:** An Explanation and Elaboration article discusses each checklist item and gives methodological background and published examples of transparent reporting. The STROBE checklist is best used in conjunction with this article (freely available on the Web sites of PLoS Medicine at http://www.plosmedicine.org/, Annals of Internal Medicine at http://www.annals.org/, and Epidemiology at http://www.epidem.com/). Information on the STROBE Initiative is available at www.strobe-statement.org.
